# Generation of Stable Lipid Raft Microdomains in the Enterocyte Brush Border by Selective Endocytic Removal of Non-Raft Membrane

**DOI:** 10.1371/journal.pone.0076661

**Published:** 2013-10-04

**Authors:** E. Michael Danielsen, Gert H. Hansen

**Affiliations:** Department of Cellular and Molecular Medicine, the Panum Institute, Faculty of Health Sciences, University of Copenhagen, Denmark; University College London, United Kingdom

## Abstract

The small intestinal brush border has an unusually high proportion of glycolipids which promote the formation of lipid raft microdomains, stabilized by various cross-linking lectins. This unique membrane organization acts to provide physical and chemical stability to the membrane that faces multiple deleterious agents present in the gut lumen, such as bile salts, digestive enzymes of the pancreas, and a plethora of pathogens. In the present work, we studied the constitutive endocytosis from the brush border of cultured jejunal explants of the pig, and the results indicate that this process functions to enrich the contents of lipid raft components in the brush border. The lipophilic fluorescent marker FM, taken up into early endosomes in the terminal web region (TWEEs), was absent from detergent resistant membranes (DRMs), implying an association with non-raft membrane. Furthermore, neither major lipid raft-associated brush border enzymes nor glycolipids were detected by immunofluorescence microscopy in subapical punctae resembling TWEEs. Finally, two model raft lipids, BODIPY-lactosylceramide and BODIPY-GM_1_, were not endocytosed except when cholera toxin subunit B (CTB) was present. In conclusion, we propose that constitutive, selective endocytic removal of non-raft membrane acts as a sorting mechanism to enrich the brush border contents of lipid raft components, such as glycolipids and the major digestive enzymes. This sorting may be energetically driven by changes in membrane curvature when molecules move from a microvillar surface to an endocytic invagination.

## Introduction

The brush border of small intestinal enterocytes is a highly specialized cell membrane optimized for providing the organism with a maximal digestive and absorptive capacity for dietary nutrients [1]–[3]. Its microvillar organization is defined by an inner actin cytoskeleton core connected to the membrane by cross filaments, and just below the brush border, each actin filament is anchored to a myosin-rich region called the terminal web, providing physical stability and possibly a contractile ability to the whole brush border [2], [4], [5]. To withstand the harsh environment in the gut lumen owing to the presence of pancreatic digestive enzymes, bile salts and microorganisms, the lipid composition of the brush border has an unusually high percentage of glycolipids, which in the case of the pig exceedes 30% [6]. Glycolipids, together with cholesterol and sphingomyelin, are known to spontaneously promote formation of liquid-ordered microdomains, commonly known as lipid rafts, in the exoplasmic leaflet of the cell membrane [7], [8]. Whereas in other cell types lipid rafts are generally considered to be small and dynamic, those of the brush border are thought to be relatively large and stable [9]. Thus, a biphasic distribution of membrane thickness has been reported for microvillus membranes with domains of increased thickness, proposed to represent lipid raft microdomains, having a lower size limit of 600 nm^2^ [10]. The lipid raft stability is owed at least partly to the abundant presence of glycolipids and lectins, including members of the galectin family [11] and intelectin, which are capable of cross-linking lipids and proteins [12]. In addition, lectin-like antiglycosyl antibodies deposited in the brush border may help protecting against luminal pathogens [13], [14].

Membrane trafficking in polarized epithelial cells such as enterocytes is a complex network of pathways operating to generate and maintain the asymmetry of the cell membrane [15]–[19]. Sorting of basolateral- and apical membrane components to their respective domains is an essential feature of this system which relies on a variety of different molecular signals together with a cellular machinery along the secretory pathway to decode them. Signals for apical sorting have proved to be very diverse in nature and those hardest to identify, but today lipid rafts are commonly thought to act as lateral sorting platforms for apical-destined cargo proteins [20]. Nevertheless, direct evidence that raft lipids are actually enriched in apical transport carriers is still scarce [20], and a non-raft pathway to the apical cell surface has also been reported [21]. In this context, it is hard to envisage how apical sorting along the biosynthetic pathway alone can account for the high enrichment of glycolipids in the intestinal brush border. With regard to endocytic membrane traffic, the brush border is generally believed to be very restrictive after weaning when uptake of macromolecules, primarily maternal immunoglobulins, ceases abruptly in a process known as “closure” [22]. Nevertheless, in a previous work, a constitutive endocytic pathway was shown to operate in enterocytes of cultured jejunal mucosal explants by use of the fluorescent lipophilic FM dye [23]. Here, a characteristic labeling of early endosomes in the terminal web region (hence called “TWEEs”) was observed to persist for periods up to 1 h without further progression deeper into the cytoplasm. It was proposed that the actomyosin cytoskeleton of the terminal web inhibited further penetration by acting as an intracellular permeability barrier for the TWEEs.

In the present work, the endocytic uptake into the TWEEs described above was characterized in further detail. Altogether, the data show that unlike FM and the polar tracer Lucifer yellow (LY), typical lipid raft-associated components of the brush border, such as the major digestive enzymes and glycolipids, were not taken up into TWEEs. Since endocytosed FM was absent from detergent resistant membranes (DRMs), the constitutive endocytosis serves to remove selectively non-raft membrane from the brush border. We propose this to be a novel sorting mechanism whereby the brush border becomes iteratively enriched in the lipid raft components necessary for formation of its unique architecture. Sorting in a polarized cell therefore need not occur solely in the intracellular compartments of the secretory pathway; it may take place also at the final destination at the cell surface.

## Materials and Methods

### Materials

N-(4, 4-difluoro-5, 7-dimethyl-4-bora-3a, 4a-diaza-s-indacene-3-pentanoyl) sphingosyl 1--D-lactoside (BODIPY FL C_5_-lactosylceramide) complexed to bovine serum albumin, BODIPY FL C_5_-ganglioside GM_1_ complexed to bovine serum albumin, Lucifer yellow CH ammonium salt, FM lipophilic styryl dye (FM 1-43 FX), Alexa-conjugated phalloidin, Cascade Blue-conjugated dextran (molecular mass ∼10.000), Lysotracker Red DND-99, Alexa-conjugated cholera toxin subunit B, Alexa-conjugated secondary antibodies for immunofluorescence microscopy, and ProLong antifade reagent with DAPI were obtained from Invitrogen (www.invitrogen.com), horseradish peroxidase-conjugated swine anti-rabbit IgG immunoglobulins and rabbit antibodies to human IgA and apoA-1 from DAKO (www.dako.com), rabbit antibodies to intestinal alkaline phosphatase from AbD Serotec (www.biogenesis.co.uk/), and Ruthenium red from Sigma-Aldrich (www.sigmaaldrich.com). Rabbit antibodies to pig intestinal lactase/phlorizin hydrolase (LPH) and to human intestinal brush border enzymes were described previously [24], [25].

### Animals

All animal experimentation in Denmark is subject to ethical evaluation by the Ministry of Justice’s Council for Animal Experimentation. No experiments with animals which involves pain, suffering, distress or lasting harm equivalent to, or higher than, that caused by the introduction of a needle in accordance with good veterinary practice may be performed without this ethical evaluation and license. All animal experimentation included in this work was performed under license 2012-15-2934-00077.

Segments of jejunum, taken about 2 m from the pylorus of overnight fasted, post-weaned pigs, were surgically removed from the anaesthetized animals by licensed staff at the department of Experimental Medicine, the Panum Institute, University of Copenhagen. After obtaining the intestinal tissue, the animals were sacrified by an injection with pentobarbital/lidocaine (1 mg/kg bodyweight).

### Organ culture of jejunal mucosal explants

Jejunal segments of about 20 cm in length were quickly removed from the animals and placed in ice-cold RPMI medium. Mucosal explants of ∼0.1 g were excised with a scalpel and cultured in RPMI medium at 37°C for periods of 0.5–1 h, essentially as described previously [26]. The various probes for fluorescence- or electron microscopy were used at the following concentrations in the culture medium: Lucifer yellow (LY): 1 mg/ml, FM: 20 µg/ml, BODIPY-lactosylceramide and BODIPY-GM_1:_ 0.1 mg/ml, Lysotracker: 10 µM, Ruthenium Red (RR): 0.2% (w/v). After culture, the explants were quickly rinsed in fresh medium and immersed in fixative at 4°C.

### Fluorescence microscopy

Cultured mucosal explants were fixed for 2 h or overnight at 4°C in 4% paraformaldehyde in 0.1 M sodium phosphate, pH 7.2 (buffer A). After a rinse three times in buffer A, the tissue was immersed overnight in 25% sucrose in buffer A before mounting and sectioning at –19°C in a Leica CM1850 cryostat. For immunolabeling, sections were incubated for 1 h at room temperature with antibodies to brush border enzymes, apoA-1, or IgA (both diluted 1∶100) in 50 mM Tris-HCl, 150 mM NaCl, 0.5% ovalbumin, 0.1% gelatin, 0.2% teleostean gelatin, 0.05% Tween 20, pH 7.2 (buffer B), followed by incubation for 1 h at room temperature with the appropriate Alexa-conjugated secondary antibodies (1∶200 dilution in buffer B). Controls with omission of primary antibodies were routinely included in the immunolabeling experiments. For labeling with fluorescent probes, sections were incubated with Alexa-conjugated phalloidin (1 U/ml, 1 h) or Alexa-conjugated CTB (10 µg/ml, 2 h at room temperature or overnight at 4°C).

All sections were finally mounted in antifade mounting medium with DAPI and examined in a Leica DM 4000B microscope fitted with a Leica DFC495 digital camera.

### Electron microscopy

Mucosal explants cultured in the presence of RR were fixed for 20 h at 4°C in 3% (v/v) glutaraldehyde, 2% (w/v) paraformaldehyde in buffer A containing 0.2% (w/v) Ruthenium red, followed by post-fixation for 2 h at 4°C in buffer A containing osmium tetroxide 1% (w/v) and 0.3% (w/v) RR, as described previously [27]. The tissue was then treated with 1% (w/v) uranyl acetate for 1 h at room temperature, dehydrated in acetone, and finally embedded in Epon. Ultrathin sections were cut in a Pharmacia LKB Ultratome III, using a diatome diamond knife. The sections were stained with lead citrate and finally examined in a Zeiss EM 900 electron microscope fitted with a Mega View II digital camera.

### Isolation of microvillar membrane vesicles

Closed, right-side-out microvillar membrane vesicles were prepared by the divalent cation precipitation method from mucosa scraped from jejunal segments [28]. Briefly, homogenization was performed with a Potter-Elvehjem homogenizer in 10 volumes of 2 mM Tris-HCl, 50 mM mannitol, pH 7.1, containing 10 µg/ml aprotinin and leupeptin. After centrifugation at 500 *g*, 5 min, MgCl_2_ was added to the supernatant to a final concentration of 10 mM. After 10 min on ice, the homogenate was centrifuged at 1.500 *g*, 10 min. The supernatant was collected and centrifuged at 48.000 *g*, 30 min, to yield a pellet of microvillar membrane vesicles.

### Detergent resistant membrane (DRM) analysis of membrane-associated FM

This analysis was performed both with mucosal explants cultured for 1 h in the presence of FM, as well as with isolated microvillar membrane vesicles incubated in the presence of FM.

Mucosal explants were homogenized with a Potter-Elvehjem homogenizer in 2 ml HEPES-HCl, 150 mM NaCl, pH 7.1 (HEPES buffer), containing 10 µg/ml aprotinin and leupeptin. The homogenate was centrifuged at 250 *g*, 5 min, and the supernatant collected and centrifuged at 20.000 *g*, 30 min, to yield a pellet of total mucosal membranes. The pellet was resuspended in 2 ml HEPES buffer and divided into two samples of 1 ml. One sample was solubilized by addition of 1% (w/v) Triton X-100 for 10 min on ice, followed by centrifugation at 20.000 *g*, 30 min, to yield a pellet of mucosal DRMs. The second sample was processed in parallel without addition of Triton X-100, yielding a pellet of total mucosal membranes.

Microvillar membrane vesicles, resuspended in 1 ml HEPES buffer, were incubated for 15 min at 37°C in the presence of 0.1 mg/ml FM. After incubation, the suspension was divided in two samples of 0.5 ml. A pellet of microvillar DRMs was then prepared from one sample and a pellet of total microvillar membranes from the second sample, as described above for the mucosal explants. DRMs and total membranes prepared both from mucosal explants and microvillar membrane vesicles were resuspended in 50 µl HEPES buffer and dilution series of 1, 1/2,1/4, 1/8 and 1/16 were prepared with HEPES buffer. Finally, samples of 2- or 5 µl of the dilution series were spotted onto Whatman filter paper and images were captured under uv-light. Samples of membranes similarly prepared were subjected to SDS/PAGE in 10% gels. After electrophoresis and electrotransfer onto PVDF membranes, successive immunoblottings were performed with antibodies to intestinal alkaline phosphatase (AP) and to lactase/phlorizin hydrolase (LPH), followed by incubation with horseradish peroxidase-conjugated secondary antibodies. Blots were developed with an electrochemiluminescence reagent using a protocol supplied by the manufacturer (GE Healthcare, www.gehealthcare.com). After immunoblotting, total protein was visualized by staining with Coomassie brilliant blue R250 (0.2% dissolved in an ethanol/H_2_O/acetic acid mixture (50:53:7).

## Results

### Apical endocytosis probed with lipophilic and polar tracers

**Figure 1:**
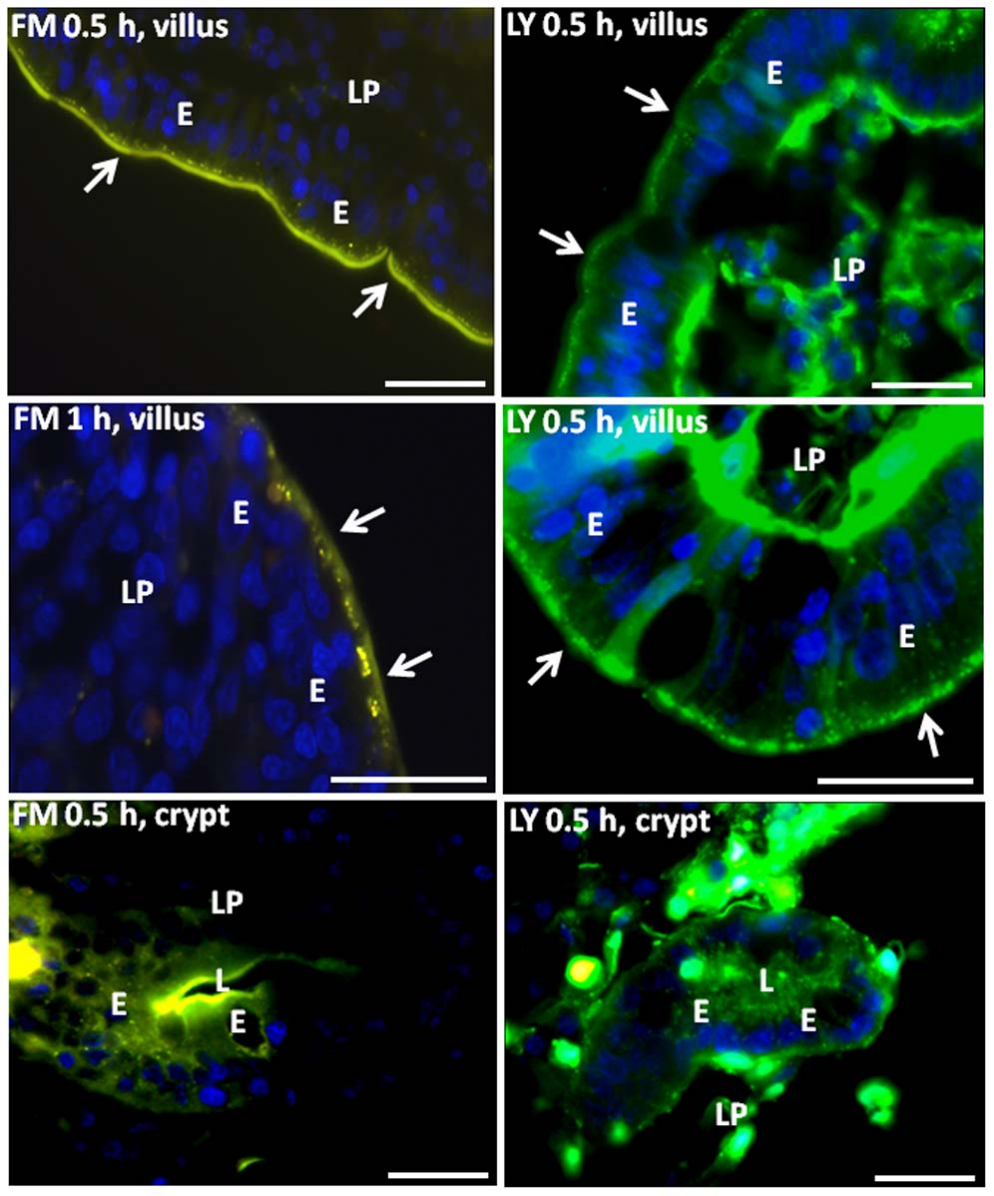
Apical endocytosis probed with FM and LY. Mucosal explants were cultured for 0.5- or 1 h in the presence of either FM (left panel images) or LY (right panel images), as described in Methods. At 0.5 h, the lipophilic FM was mainly seen in the brush border (arrows) of villus enterocytes (E), but also labeled the subapical TWEEs, whereas at 1 h, most of the dye was seen in the latter compartment. No labeling was seen along the lateral surfaces or in the lamina propria (LP), indicating that FM did not pass through the tight junctions. The polar LY did not stain the brush border, but like FM, it distinctly labeled the subapical punctae of TWEEs. In addition, LY strongly labeled the lamina propria (LP) and faintly the lateral surfaces, indicating a passage through the tight junctions. In the crypt cells, both FM and LY visualized numerous punctae scattered in the cytoplasm. Bars, 20 µm.

Except for a short neonatal period until endocytic uptake of undigested dietary macromolecules abruptly ceases [22], [29], the brush border of villus enterocytes acts as a permeability barrier. Nevertheless, using a jejunal organ culture system and the water-soluble, fixable lipophilic fluorescent FM dye, a constitutive apical endocytosis can be visualized (Figure 1, left panel images) [23]. Whereas endocytosis in most cells progresses from early endosomes via late endosomes to lysosomes on the minute scale [30], early endosomes are retained in the subapical terminal web region of villus enterocytes (hence termed “TWEEs” [23]) for periods up to 1 h or more. This strikingly narrow localization contrasts with the wide intracellular distribution of fluorescent punctae seen in immature crypt cells of the same tissue sections (Figure 1).

To characterize apical endocytosis in more detail, we employed the non-toxic fluorescent polar tracer Lucifer yellow (LY) [31], and as shown in Figure 1, right panel images, the TWEEs labeled by FM were also distinctly LY-positive. In addition, with a molar mass of 444 g/mol, LY was sufficiently small to penetrate the tight junctions between enterocytes and accumulated in the underlying lamina propria. In contrast to LY, soluble tracers of higher molecular mass, such as dextran (10.000) or horseradish peroxidase (44.000) were not observed to be taken up in TWEEs (data not shown). Thus, a significant uptake of fluid phase components into TWEEs occurs in a constitutive manner, but only of small, metabolite-size molecules.

### Brush border enzymes and glycolipids are absent from TWEEs

**Figure 2:**
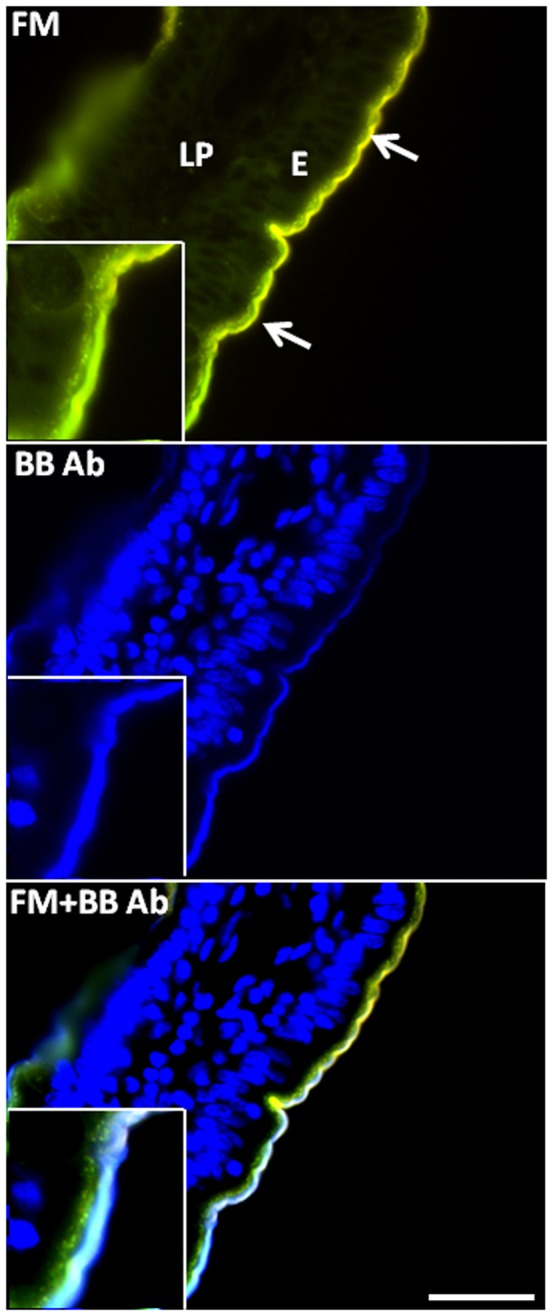
Absence of brush border enzymes from TWEEs. A section of a mucosal explant cultured for 0.5 h with FM. The section was labeled by incubation with an antibody raised to an isolated brush border fraction, followed by labeling with an Alexa Fluor 350-conjugated secondary antibody (blue color). Like FM, the antibody labeled the brush border (arrows) of the enterocytes (E) but not the subapical TWEEs, indicating that none of the major brush border enzymes have been endocytosed during culture. Inserts show parts of the images at a two-fold higher magnification. Nuclei were visualized in blue color by staining with DAPI. Bar, 20 µm.

Figure 2 shows a villus section of a mucosal explant after 30 min of exposure to FM. At this time point, both the brush border and the underlying TWEEs were labeled. Incubation of the section with an antibody raised to a purified brush border fraction [25] only labeled the apical surface, but not the newly formed TWEEs. This particular antibody recognizes a large number of major microvillar hydrolases (glycosidases, peptidases, alkaline phosphatase), and the result thus indicates that none of these proteins were internalized into TWEEs. This is in line with a previous observation that ferritin associated with brush border glycoproteins was not taken up by endocytosis in cultured human mucosal biopsies [32].

**Figure 3:**
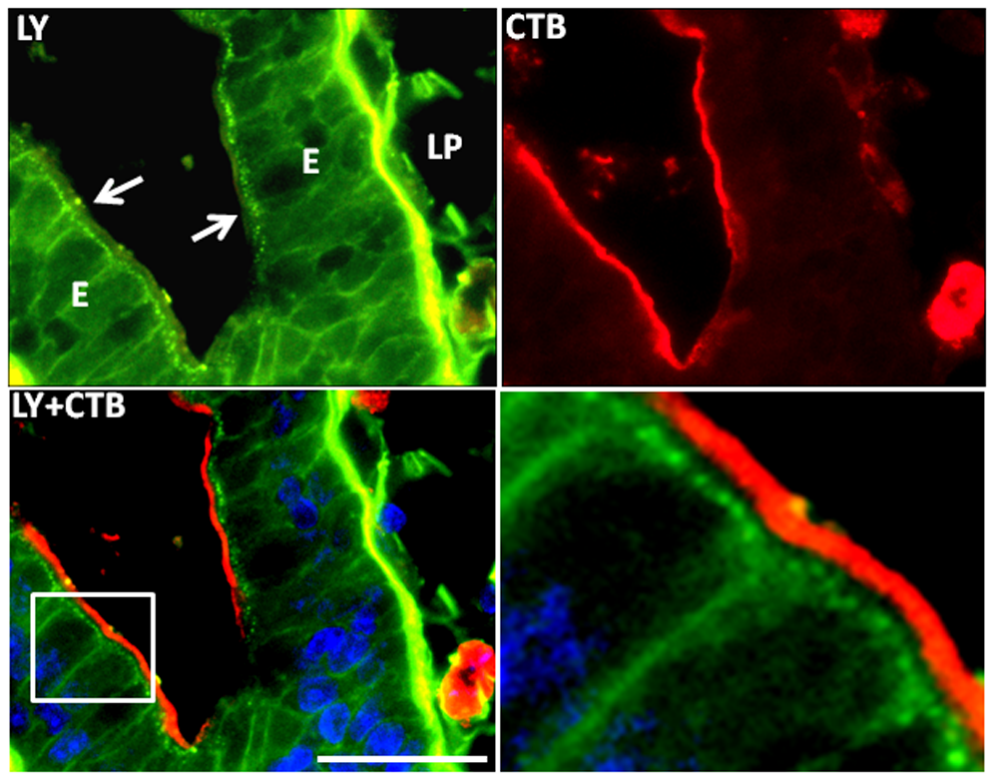
Absence of GM_1_ from TWEEs. A section of a mucosal explant cultured for 0.5 h with LY and labeled with Alexa-conjugated CTB. LY distinctly visualized the subapical TWEEs and the basolateral surface of the enterocytes (E), as well as the lamina propria (LP). CTB strongly labeled the brush border (arrows) but was not detectable in the TWEEs, indicating that GM_1_ has not been endocytosed during culture. (The boxed part of the merged image is shown separately at a three-fold higher magnification). Bar, 20 µm.

**Figure 4:**
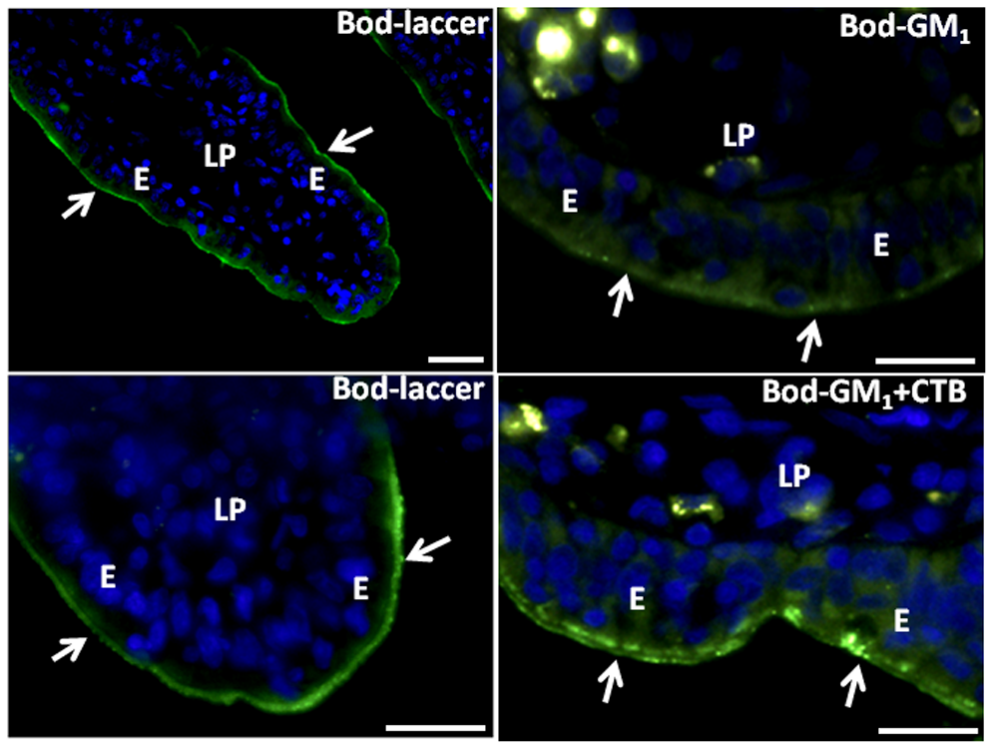
Absence of BODIPY-glycolipids from TWEEs. Sections of mucosal explants cultured for 1 h in the presence of BODIPY-lactosylceramide (Bod-laccer), BODIPY-GM_1_ (Bod-GM_1_), or BODIPY-GM_1_ and CTB (Bod-GM_1_+CTB). BODIPY-lactosylceramide strongly labeled the brush border (arrows), but distinct subapical punctae indicative of TWEEs were not detected. BODIPY-GM_1_ alone only weakly labeled the brush border and not the TWEEs, but in the presence of CTB a distinct, patchy labeling of the brush border was seen. In addition, the subapical TWEEs were prominently labeled. Bars, 20 µm.

Figure 3 shows a similar labeling experiment, using LY instead of FM, and it clearly demonstrates that ganglioside GM_1_, visualized by its ligand Alexa-conjugated CTB, remained at the apical cell surface during the formation of the LY-positive TWEEs. Fluorescent BODIPY derivatives of sphingolipids, including lactosylceramide and ganglioside GM_1_, have been widely used previously to study membrane traffic and membrane microdomains of living cells [33], [34], and the binding and internalization of exogenously added BODIPY-lactosylceramide and BODIPY-GM_1_ are shown in Figure 4. After 1 h of culture, the former strongly and uniformly bound to the brush border. The labeling had a characteristic “knobbly” appearance, suggestive of a heterogeneous membrane distribution, but no internalization into TWEEs was detectable. In a parallel experiment and at the same concentration, the binding of BODIPY-GM_1_ to the brush border by comparison was much weaker and more patchy, but as with lactosylceramide no distinct uptake into TWEEs was observed. CTB has previously been shown to increase significantly the endocytosis from the brush border [35], and when added together with this BODIPY-GM_1_, it greatly increased the glycolipid binding and by 1 h much of it had been taken up into TWEEs, showing that the fluorescent glycolipid acted as surface receptor for the toxin in an authentic manner (Figure 4).

Taken together, the above results show that neither the major digestive enzymes of the brush border nor two prominent glycolipids are taken up by the constitutive endocytosis visualized by LY and FM. Since both types of membrane constituents are continually delivered to the brush border by exocytosis, the selective exclusion from TWEEs will result in their gradual accumulation in the brush border during the life span of the enterocyte.

### DRM analysis of membrane-associated FM

**Figure 5:**
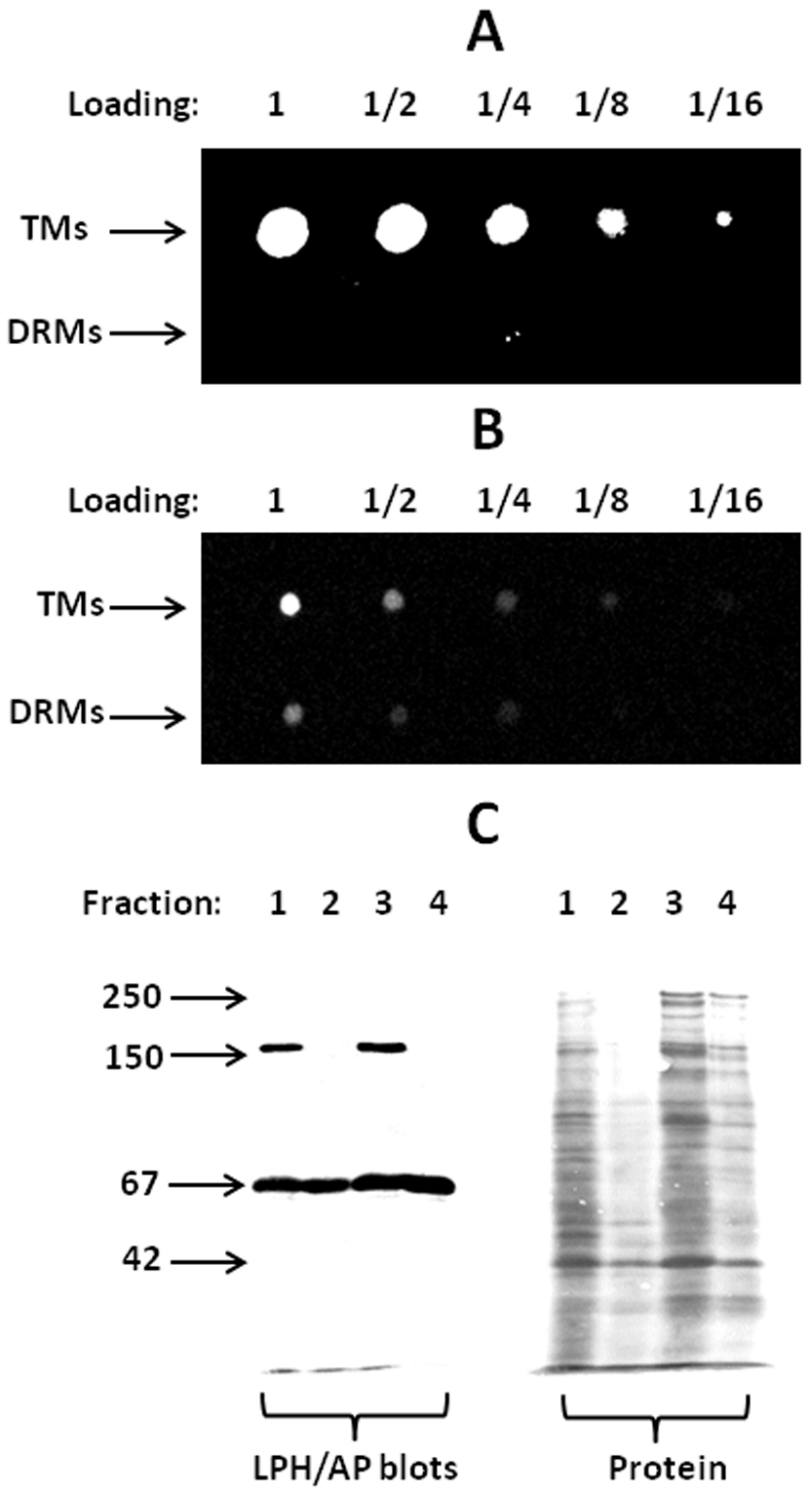
DRM analysis of membrane-associated FM. Total membranes (TMs) and DRMs were prepared from mucosal explants cultured for 1 h in the presence of FM (A) or from microvillar membrane vesicles treated with FM (B), as described in Methods. 5 µl (A) -or 2 µl (B) samples of serial dilutions of the membrane fractions were spotted onto Whatman filters and images captured under uv-light. FM was detected in TMs of both mucosal explants and microvillar membrane vesicles, but only in DRMs of the latter. C: SDS/PAGE of TMs and DRMs prepared as described in Methods from either total mucosal- or microvillar membranes. Samples of 25 µl were applied to each well. 1: Mucosal TMs; 2: Mucosal DRMs; 3: Microvillar TMs; 4: Microvillar DRMs. After electrophoresis and transfer onto a PVDF membrane, the lipid raft marker alkaline phosphatase (AP, 67 kDa) and lactase/phlorizin hydrolase (LPH, 160 kDa), a non-raft marker, were visualized by immunoblotting. Total protein was stained with Coomassie brilliant blue. Molecular mass values (kDa) are indicated by arrows.

**Figure 6:**
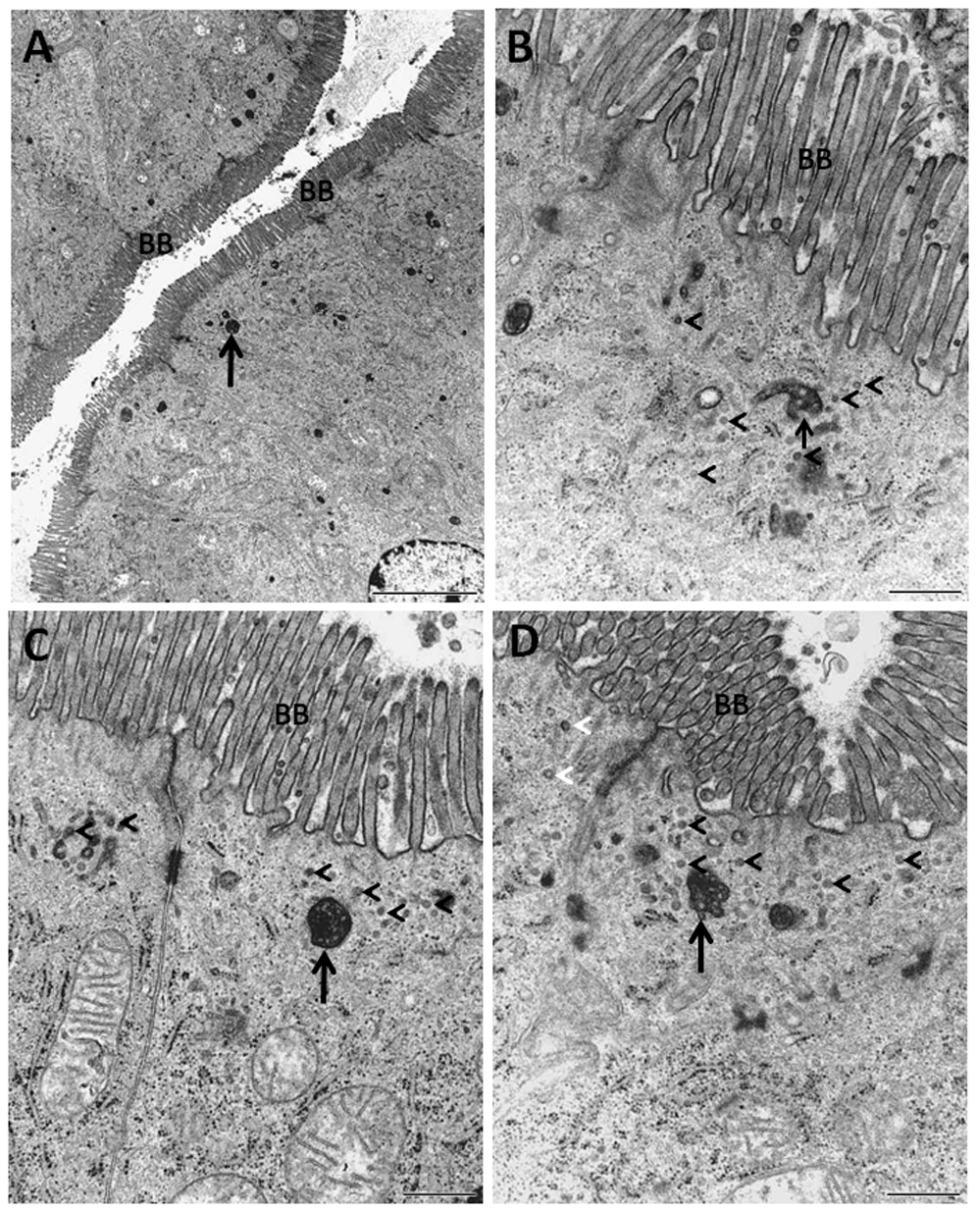
Apical endocytosis probed with Ruthenium red (RR). Electron micrographs of sections of mucosal explants cultured for 1 h in the presence of RR. A: At low magnification, electron-dense RR was seen all along the brush border (BB). B-D: At higher magnification, RR visualized subapical multivesicular bodies (arrows) surrounded by numerous small vesicle-like structures, putatively identified as TWEEs (arrowheads). Bars, 5 µm (A); 0.5 µm (B-D).

Detergent resistant membranes (“DRMs”) are thought to be the biochemical equivalent of lipid raft microdomains [36], [37]. As shown in Figure 5A, none of the FM that associated with total membranes from mucosal explants, labeled with the dye for 1 h, was detected in the corresponding DRM fraction, using Triton X-100 as detergent. Since at this time point most of the FM was seen in TWEEs (Figure 1), the experiment implies that apical FM-positive early endosomes are mainly composed of detergent sensitive membranes, i.e., non-raft- or liquid disordered membranes [38]. Figure 5B shows a similar DRM analysis performed with a microvillar membrane vesicle fraction directly exposed to FM. In this experiment the dye was likewise clearly seen in the total membranes, but in addition it was also detectable in the DRM fraction, indicating that FM spontaneously inserts into both raft- and non-raft microdomains of the brush border. Together, the results of the above experiments imply that FM is partially redistributed from raft- to non-raft microdomains before its internalization into TWEEs. As a control experiment to validate this conclusion, the distribution of the GPI-linked lipid raft marker AP in the membrane fractions is shown in Figure 6C. For both total mucosal- and microvillar membranes, the 67 kDa band of AP was equally prominent in both TM- and DRM fractions, indicating that lipid rafts were efficiently recovered in the latter. In contrast, the 160 kDa lactase/phlorizin hydrolase (LPH), the only major microvillar hydrolase predominantly residing in the non-raft part of the membrane [21], [39], was absent from the DRM fractions. In addition, staining with Coomassie brilliant blue revealed that only a minor part of the total protein was present in the DRM fractons.

### Morphological characterization of TWEEs

**Figure 7:**
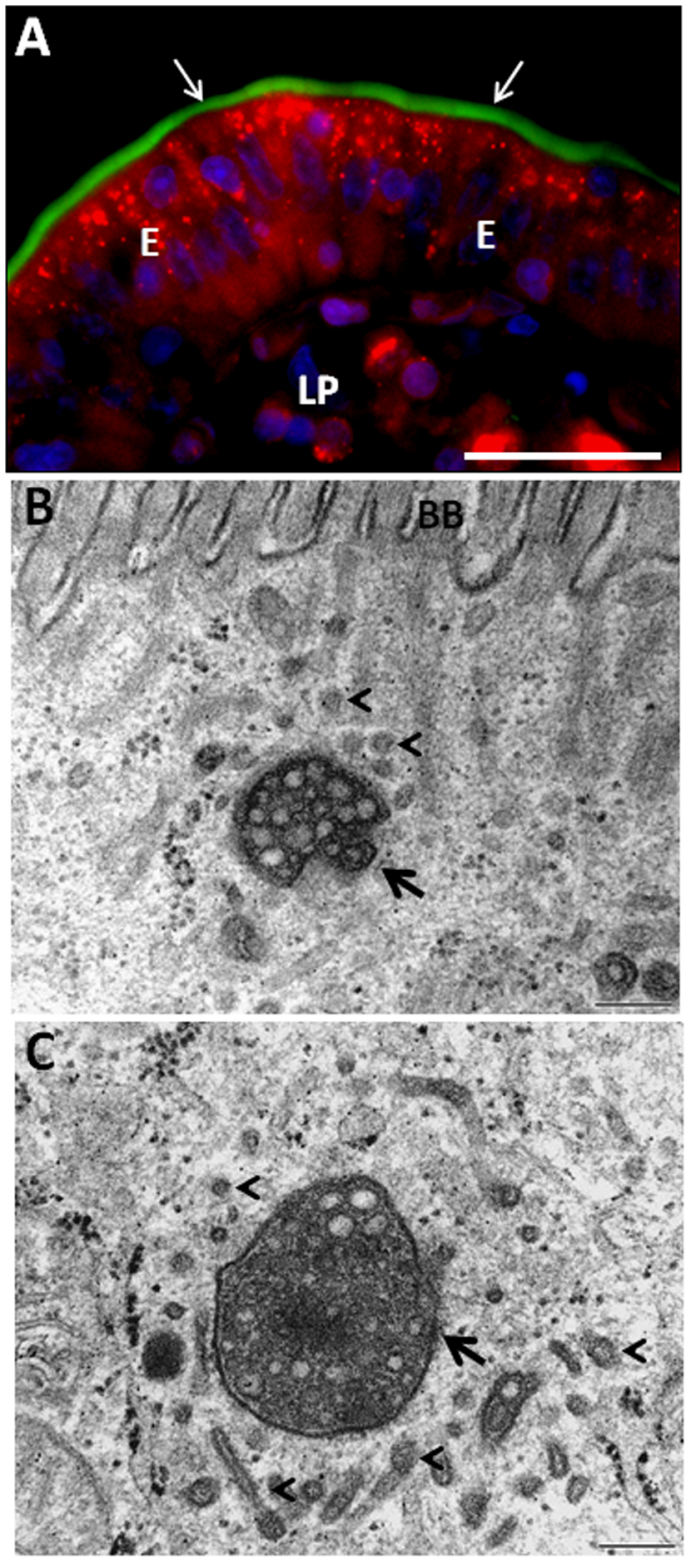
Acidifying organelles identified with Lysotracker. A: Section of a mucosal explant labeled for 1 h with red Lysotracker as described in Methods. Punctae, representing acidic organelles (lysosomes, late endosomes, MVBs) were scattered throughout the cytoplasm of the enterocytes (E), including the subapical region below the brush border (arrows) visualized by green phalloidin. B-C: Close-up electron micrographs of sections of RR-exposed mucosal explants, as described in the legend to Figure 6. MVBs (arrows) were present just below the brush border (BB) surrounded by TWEEs. Some of the latter seemed to be undergoing tubulation (arrow heads). LP, lamina propria. Bars, 20 µm (A); 0.2 µm (B, C).

Ruthenium red (RR) is a membrane-impermeable, electron-dense membrane marker of low molar mass (786 g/mol) suitable for transmission electron microscopy [40]. When applied to mucosal explants during tissue fixation, we have previously used RR to probe for cell – and tight junction integrity [27] and for visualization of surface-connected deep apical tubules in the brush border [41]. In the present work RR was added to the medium during culture for 1 h, similarly to FM and LY, and as shown in Figure 6A, the dye uniformly labeled the entire microvillar surface. Inside the enterocytes, multivesicular bodies (MVBs) of about 0.3–0.5 µm, mostly spherical but some with tubular extensions, were distinctly labeled in the subapical terminal web region, indicating an endocytic uptake of RR from the brush border. At higher magnification, numerous RR-positive smaller vesicular structures, of about 50–100 nm in diameter, were also observed in the terminal web region, often in close proximity to MVBs (Figure 6B-D, Figure 7B-C). Based on the similar narrow subapical distribution, we take these vesicle-like structures to represent the FM- and LY-positive TWEEs visualized by fluorescence microscopy.

Overall, the above experiments with RR show that subapical MVBs are generated, at least in part, by apical endocytosis followed by fusion with the newly formed TWEEs. Although multiple functions have been ascribed to MVBs, their main role is to serve as a portal for degradation of membrane constituents in the lysosomes [42]. Figure 7A shows a labeling with Lysotracker, a fluorescent acidotrophic probe, and as can be seen, acidic organelles were scattered throughout the cytoplasm of the enterocytes. Occasionally they were localized in proximity of the brush border, indicating that the MVBs in the terminal web region are indeed acidifying organelles. This finding agrees well with our previous observation that FM-positive punctae colocalize with the early endosome marker EEA-1 in the apical cytoplasm [23]. Therefore, TWEEs most likely represent an intermediate stage in a transport mechanism leading to an intracellular breakdown of membrane from the brush border.

### TWEEs are not a hub in exocytosis or transcytosis

**Figure 8:**
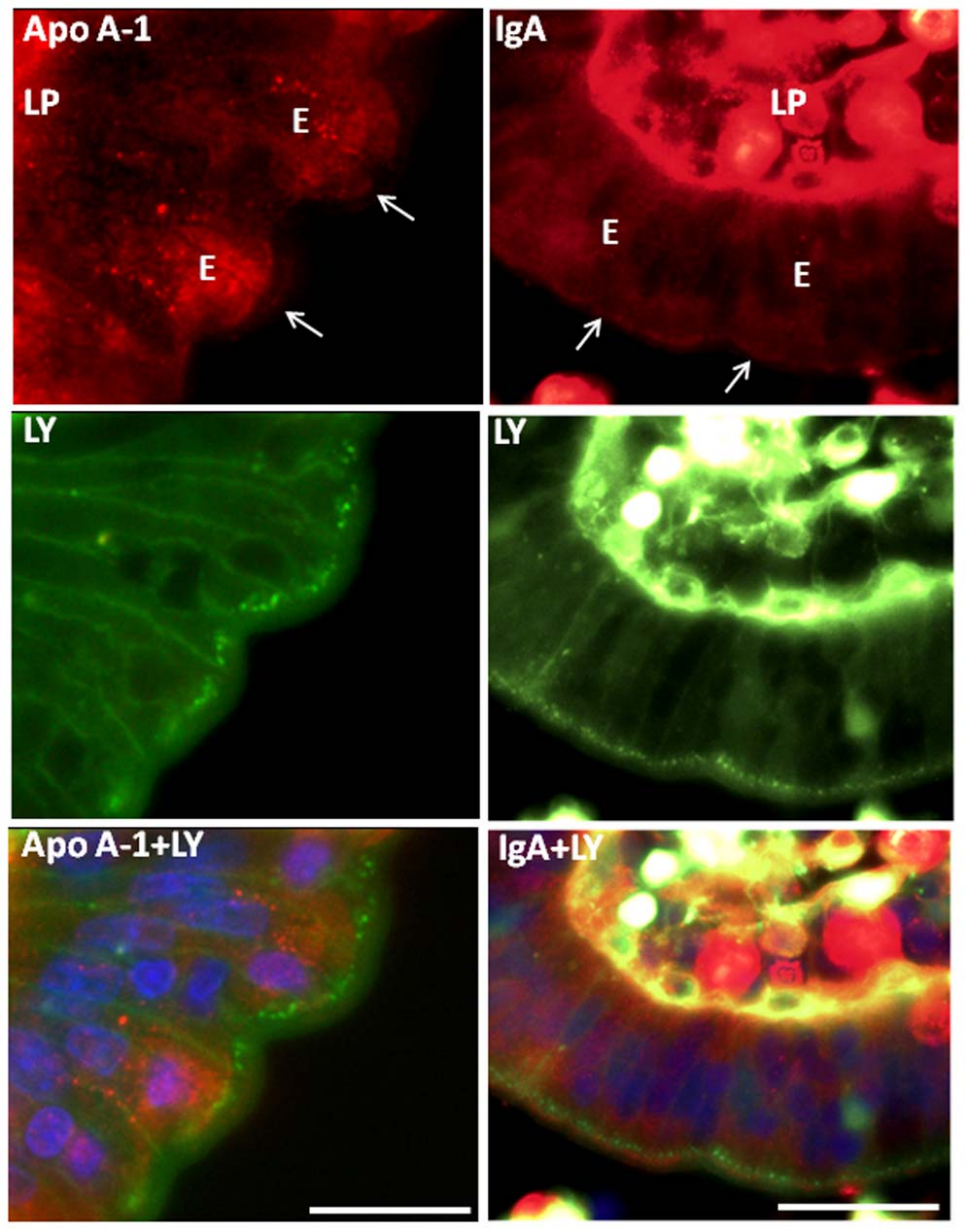
Absence of apoA-1 and IgA from TWEEs. Sections of mucosal explants cultured for 1 h in the presence of LY and labeled with antibodies to apoA-1 (left panel images) or IgA (right panel images). ApoA-1 was seen faintly in patches at the brush border (arrows) and in numerous punctae scattered in the cytoplasm, but not in the LY-positive TWEEs. Intense labeling for IgA was seen in plasma cells in the lamina propria (LP). In the enterocytes (E), a diffuse labeling was seen throughout the cytoplasm as well as in the brush border, but not in the TWEEs visualized by LY. Bars, 20 µm (A).

Due to their very long residency in the subapical region, TWEEs might also function as a hub in the exocytic membrane trafficking. To explore this possibility, sections of explants labeled for 1 h with LY were immunostained with antibodies to either apoA-1 or IgA. ApoA-1 is constitutively secreted apically from the enterocytes [27], [43], and as shown in Figure 8, left panel images, some intracellular punctate compartments and, more weakly, the apical surface were visualized by the antibody. However, no colocalization with LY-positive TWEEs was detected, indicating that the latter are not a hub for apoA-1 en route to the apical cell surface. IgA synthesized by plasma cells in the lamina propria is taken up at the basolateral membrane of the enterocytes by receptor-mediated endocytosis and subsequently transcytosed to the opposite pole of the cell for discharge into the gut lumen [44]–[46]. In the enterocytes, the anti-IgA antibody resulted in a widespread, diffuse labeling in the cytoplasm as well as of the brush border (Figure 8, right panel images). But as with apoA-1, the lack of colocalization with LY implies that TWEEs are not part of the itinerary for the basolateral-to-apical transcytosis.

In summary, our results show that TWEEs, previously observed to be formed constitutively at the brush border, serve to selectively remove non-raft membrane constituents from the brush border. Rather than acting as a hub for apical membrane trafficking, their destiny primarily appears to enter a degradative pathway via subapical MVBs towards the lysosomes.

## Discussion

In a previous work, the lipophilic membrane probe FM identified a constitutive endocytosis from the porcine jejunal brush border into a distinct population of early endosomes localized in a narrow band about 1–2 µm below the apical cell surface [23]. Thus named TWEEs, as this part of the enterocyte is known as the terminal web region due to the dense meshwork of actomyosin filaments [1], [47], we proposed that their accumulation and residency here for periods up to 1 h or more was part of the overall permeability barrier of the gut [23]. The observation of the present work that the polar tracer LY labeled the TWEEs similarly to FM demonstrates that the constitutive apical endocytosis is a *bona fide* process and not an event artefactually induced by membrane incorporation of the dye. The failure of larger fluid phase probes such as 10 kDa-dextran to be taken up into TWEEs most likely reflects that this endocytosis occurs after “closure” in post-weaned animals at a time when nutrient macromolecules are degraded intraluminally [48]. Like LY, the electron-dense probe RR was sufficiently small also to be internalized, and it revealed the TWEEs to be a uniform population of 50–100 nm-sized vesicle-like structures. Strongly RR-labeled MVBs, likewise localized in the terminal web region, indicated that the TWEEs most likely are destined ultimately for degradation in the lysosomes [42]. However, labeled tubulo-vesicular structures, possibly representing apical recycling endosomes [15], [19], [49], were also occasionally observed, suggesting that some membrane components internalized by TWEEs may be returned to the brush border. The observation that neither apoA-1 (an exocytic marker) nor IgA (a transcytic marker) detectably colocalized with LY implies that these membrane trafficking routes bypass the TWEEs. In this context, the cytoskeletal meshwork of the terminal web most likely contributes by acting as a physical diffusion barrier preventing the TWEEs from interaction with other parts of the complex system of membrane trafficking pathways of mature enterocytes.

The DRM analysis of membrane-associated FM clearly indicated that although the dye incorporates into both raft- and non-raft fractions of microvillar membranes, only the latter are taken up into TWEEs in cultured explants. This finding implies that lipid raft-associated FM is excluded from the membrane endocytosed from the brush border. Furthermore, the model raft glycolipids BODIPY-lactosylceramide and BODIPY-GM_1_ were incorporated into the brush border but clearly failed to label the TWEEs, except when CTB was added simultaneously. This result contrasts strongly with similar labeling studies performed with fibroblasts where both BODIPY glycolipids were efficiently taken up into intracellular organelles [33], [34]. Therefore, in addition to the conclusions stated above, the results of the present work point to a novel role for TWEEs: As a mechanism for continuous removal of non-raft membrane components from the brush border. The consequence hereof is that proteins and lipids that preferentially associate with lipid rafts will be iteratively enriched in the brush border during the short lifespan of the enterocyte, and to our knowledge, such a counter-flow type of mechanism at the cell surface for improving the sorting efficiency of lipid raft proteins- and lipids has not previously been recognized. The generation of epithelial cell polarity is otherwise thought to occur by sorting events taking place in the biosynthetic pathway or in the complex system of endocytic compartments [15], [17]–[20]. Yet, considering that glycolipids account for almost one-third of the total membrane lipids of the porcine brush border [6], a proportion far higher than that of the average cell membrane (∼5%), selective removal of non-raft components, mainly glycerophospholipids, at the brush border may be required to achieve the extraordinary lipid composition of this membrane.

**Figure 9:**
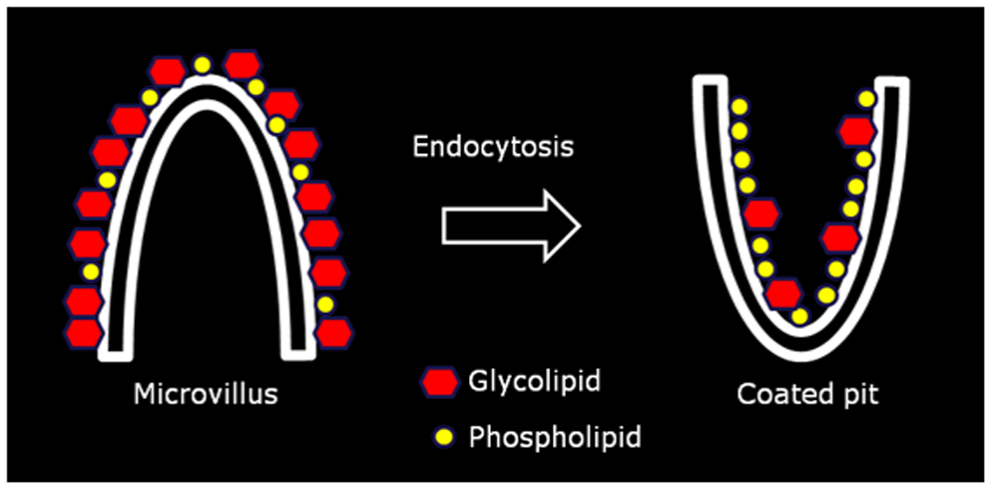
Proposed model for glycolipid enrichment in the brush border by selective endocytosis of non-raft membrane lipids. Endocytosis requires lateral molecular movement of membrane lipids from a microvillus with strong negative curvature to a coated pit with positive curvature. This movement is energetically favored by phospholipid molecules having small polar headgroups, whereas bulky glycolipids preferentially will remain in the microvillus.

An important unsolved question is how lipid raft components escape endocytosis to be retained in the brush border. In the case of the major digestive enzymes, they have either no or only short cytoplasmic tails without signals for internalization [50], but this does not necessarily prevent them from being included as cargo in a constitutive endocytosis. However, with regard to endocytosis, the microvillus architecture of the brush border means that for sterical reasons, only the small patches of apical membrane between adjacent microvilli are available for membrane invagination and subsequent vesicle formation. This process involves creating a positive curvature of the membrane, i.e. the opposite of the strong negative curvature that prevails along the microvilli. For the lipid raft-forming glycolipids with their bulky headgroups, it may simply be energetically more favourable to reside in the exoplasmic leaflet of a membrane with negative- rather than positive curvature. This view, depicted in the model shown in Figure 9, is at least consistent with the observed ability of the pentameric, negative curvature-forming toxin CTB to induce apical endocytosis of BODIPY-GM_1_ and with curvature-driven lipid sorting previously described for other membrane systems [51]–[53].
